# A Blood-based Metabolite Signature for Personalized Risk Assessment of Pancreatic Cancer

**DOI:** 10.33696/cancerimmunol.6.095

**Published:** 2024

**Authors:** Ricardo A León-Letelier, Yihui Chen, Riccardo Ballaro, Ehsan Irajizad, Kim-An Do, Anirban Maitra, Jianjun Zhang, C Max Schmidt, Johannes F. Fahrmann

**Affiliations:** 1 Department of Clinical Cancer Prevention, The University of Texas MD Anderson Cancer Center, Houston, TX 77030, USA; 2 Department of Biostatistics, The University of Texas MD Anderson Cancer Center, Houston, TX 77030, USA; 3 Department of Translational Molecular Pathology and Sheikh Ahmed Center for Pancreatic Cancer Research, The University of Texas MD Anderson Cancer Center, Houston, TX 77030, USA; 4 Department of Surgery, Indiana University School of Medicine, Indianapolis, Indiana, USA; 5 Department of Epidemiology, Richard M. Fairbanks School of Public Health, Indiana University, Indianapolis, Indiana, USA

## Commentary

Pancreatic ductal adenocarcinoma (PDAC) is the third most common cause of cancer-related deaths in the United States. Modeling studies project PDAC to be the second leading cause of cancer-related mortality by 2040 [1,2]. Poor survival rates are attributed to the overwhelming majority (>80%) of patients presenting with locally advanced or metastatic disease, which precludes surgical resection and possibility of a long-term cure. Substantive evidence supports that detection of localized disease, when patients are still amendable to surgical resection, is associated with improved survival outcomes [3,4].

**Figure 1.**
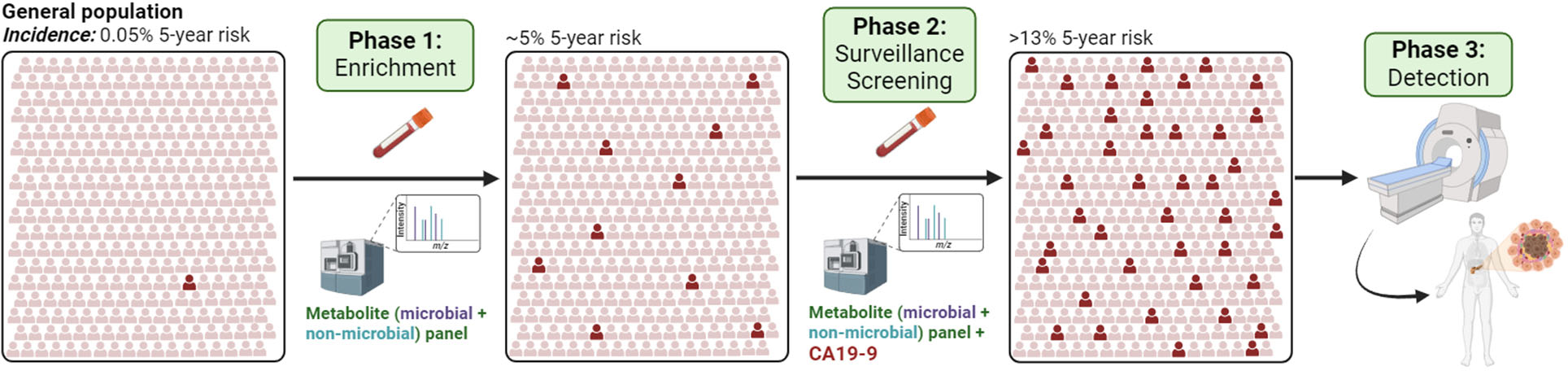
A stepwise strategy of identifying individuals at high-risk of developing PDAC (Phase 1) who would benefit from surveillance and screening (Phase 2) with pertinent cancer detection tests (e.g. CA19–9) that would trigger an imaging-based modality, such as CT/MRI, and diagnostic work-up (Phase 3) for earlier detection of PDAC. Created with Biorender.com.

The low incidence of PDAC in the general population (~13.5 per 100,000 individuals) makes it challenging to implement effective screening programs for PDAC [1]. For instance, even if a screening test has 100% sensitivity at 99% specificity, this would still result in approximately 74 false positives for every one true positive. Of note, no such test(s) yet exist that achieves these performance metrics. Presently, the United States Preventive Services Task Force (USPSTF) gave a “D” recommendation for routine PDAC screening in average-risk, citing concerns over false positives, including anxiety for the patient and risk of overdiagnosis and overtreatment, and inadequate evidence that screening or treatment of screen-detected PDAC improves disease-specific morbidity or mortality, or all cause-mortality. However, it was recognized that targeted screening in high-risk populations may be warranted [5]. Testing of high-risk individuals will increase the positive predictive value of downstream cancer screening and detection tests. To-date, several groups have been identified at high-risk of PDAC including persons with inherited risk [6,7] individuals with mucinous cystic lesions of the pancreas [8], individuals with chronic pancreatitis (CP) [9] and individuals older than 50 years of age and presenting with new-onset diabetes (NOD) [10,11]. In the context of familial pancreatic cancer (FPC), the risk level of PDAC is based on the numbers of affected family members and hereditary syndromes. For perspective, FPC relatives with three affected individuals have an estimated PDAC incidence of 288 per 100,000, which is nearly 21-fold higher incidence compared to the general population [7] International consortiums have recommended that an individual who had a 5-fold to 10-fold risk undergo PDAC screening [12]. To this end, a recent report of pancreas surveillance outcomes of high-risk individuals within the multicenter Cancer of Pancreas Screening-5 (CAPS5) study reported that five-year survival of patients with a screen-detected PDAC was 73.3% with a median overall survival of 9.8 years compared to 1.5 years for those patients diagnosed with PDAC outside of surveillance [13]. These findings suggest that screening of high-risk individuals may be effective for reducing PDAC-related mortality outcomes. Yet, the majority (~90%) of PDAC cases are sporadic without a hereditary or familial predisposition [14]. Among this population, there remains an unmet need to establish risk signatures that can identify individuals at high-risk of developing PDAC (enrichment phase) and who would benefit from surveillance and screening (detection stage) for effective earlier detection, when patients are more likely to benefit from curative intent intervention (Figure 1).

To address this need, Irajizad and colleagues developed a blood-based metabolic signature, consisting of metabolites of microbial and host origin, for predicting 5-year risk of PDAC [15]. The study leveraged 172 pre-diagnostic plasmas collected within 5 years of a PDAC diagnosis as well as 863 non-case plasma from participants in the 10 participating Prostate Lung Colorectal and Ovarian (PLCO) study centers that did not develop cancer during study follow-up. In the study, non-case controls were matched to cases 5:1 based on the distribution of age, race, sex, and calendar date of blood draw in 2-month blocks within the case cohort. Modeling of the metabolite (microbial + host) panel was predicated on the PCS (Predictability, Computability and Stability) framework [16]. Under this framework, the entire PLCO specimen set was divided into a Development Set, which was used for training and tuning model parameters (Training Set) and model selection (Validation Set), and a set-aside Test Set. In the Testing Set, the metabolite (microbial + host derived) panel achieved an age-, sex-, BMI-, and smoking status-adjusted odds ratio (OR [per unit increase]) of 3.13 (95% CI: 2.08–4.98) for 5-year risk of PDAC. Respective 5-year absolute risk estimates adjusted for prevalence of disease based on the entire intervention arm of the PLCO study based on metabolite (microbial + host) panel scores in the 80^th^, 90^th^, 95^th^, and 97.5^th^ percentiles were 1.25%, 1.89%, 2.88%, and 4.74%. The metabolite panel may thus serve as a potential tool for enriching high-risk individuals who are otherwise considered average risk (Figure 1). Importantly, the metabolite panel when combined with CA19–9, a clinically validated tumor marker for PDAC (17), improved predictive performance compared to CA19–9 alone. Individuals with a combined metabolite panel + CA19–9 score in the top 2.5^th^ percentile were found to have an absolute 5-year risk of PDAC >13%, which is sufficient risk to warrant close monitoring and would trigger an imaging-based modality such as contrast-enhanced pancreas protocol CT or MRI/MRCP [18,19].

The metabolite panel developed for predicting 5-year risk of PDAC includes three metabolites of microbial origin (trimethylamine N-oxide (TMAO); indoleacrylic acid (IAA), and an indole-derivative) that were selected by LASSO regression during modeling and that were independently associated with increased risk of PDAC. It is increasingly recognized that the microbiota plays a crucial role in human health and disease. The diversity of the microbiota is complex in each specific organ, making it a highly entangled ecosystem [20]. From an anatomical point of view, the pancreas is connected to the oral cavity, esophagus, and stomach upwards through the pancreatic duct, downward to the duodenum, and is adjacent to the common bile duct. It has been hypothesized that bacterial refluxed form the gastrointestinal tract through the pancreatic ductal may result in colonization into the pancreatic parenchyma through the papilla. Microbiota from distal sites, such as the colon, may also lead to colonization in the pancreas through other proposed pathways [21–23]. These changes may culminate in alterations in the diversity of the microbial population, namely dysbiosis [24,25], with a shift towards loss of microbial diversity and community stability that is associated with more pathogenic microbes and that may increase risk of cancer, including PDAC [23,26–27]. For example, higher abundance of oral *Porphyromonas Gingivalis*, *Aggregatibacter actinomycetemcomitans* have been linked to a higher risk of developing PDAC [28]. Changes in the tumor microbiome composition has also been linked to influencing disease progression [29]. Riquelme and colleagues reported fecal microbiota transplantation (FMT) from late-stage PDAC patients had more rapid tumor progression and significant increases in selective intratumoral bacteria such as those belonging to *Clostridium species*. This is a point of relevance given that *Clostridium species* promote production of TMAO [30]. Notably, our group also showed that elevated cystic fluid TMAO levels are associated with high-grade dysplasia and malignancy of intraductal papillary mucinous neoplasms of the pancreas [21]. Mechanistically, release of microbial-derived toxins and metabolites may result in perpetuation of inflammation and modulation of the host immune response, which contributes to oncogenesis and, more broadly, has implications in response to therapy and survival outcomes [20,31–38]. For instance, TMAO can directly promote inflammation via upregulation of CD36 and SR-A1 on macrophages, resulting in accumulation of oxidized-LDL that promotes production of inflammatory cytokines, such as TNF-α and IL-6 [39–41]. TMAO can also activate Nuclear Factor Kappa Beta (NFκB)-signaling and the nucleotide-binding domain, leucine-rich-containing family, pyrin domain-containing-3 (NLRP3) inflammasome to induce cytokine and prostaglandin production, which may promote chronic inflammation that may increase risk of PDAC [21,42–45].

Although direct evidence of indole and indole-derivatives’ involvement in PDAC is lacking, these metabolites are known ligands of the aryl hydrocarbon receptor (AHR) that is intricately involved in regulating the immune and inflammatory response as well as the pregnane X receptor (PXR) that is associated with promoting disease progression [46–48]. Hezaveh and colleagues showed that indole-mediated activation of AhR signaling in tumor-associated macrophages (TAMs) suppress anti-tumor immunity. Inhibiting AhR activation polarized TAMs towards a pro-inflammatory state that was parallelled by increased infiltration of effector memory (CD62L^neg^CD44^hi^) CD8^+^ T cells into tumors resulting in suppression of tumor growth [47]. Consequently, the occurrence of microbial metabolites in circulation may serve as a surrogate for the individuals microbial ‘health’ and susceptibility to disease.

The metabolite panel also included five host-derived metabolites that were individually associated with increased risk of PDAC: 2-hydroxyglutarate, cholesterol glucuronide, and the carbohydrates galactosamine, glucose, and erythritol. 2-Hydroxyglutarate (2HG) is a low-abundant metabolite derived from 2-oxoglutarate and exists in two enantiomeric forms, D-2HG and L-2HG. 2HG participates in cell metabolism, hypoxia signaling, and immune responses and has been associated with various malignancies, including brain, colon, breast, and PDAC [49–51]. Mutations in isocitrate dehydrogenase 1 (IDH1) and IDH2 contribute to 2HG accumulation via metabolism of α-ketoglutarate [49]. Hypoxia has also been shown to promote L-2HG production via lactate dehydrogenase A, which maintains cancer stem cells and promotes immune evasion in PDAC [52]. L-2HG was also found to be elevated in serum from patients with PDAC [52], and circulating 2HG has been proposed as a potential biomarker for IDH-mutant intrahepatic cholangiocarcinoma and cholangiocarcinoma [53,54]. Cholesterol glucuronide, a cholesterol derivative via UDP-glucuronosyltransferase (UGT), is produced in the liver [55]. The role of cholesterol glucuronide in cancer remains uncharacterized; however, UGTs are widely involved in multiple cancers, including PDAC, exerting protumor effects and drug resistance, mainly by conjugating glucuronic acid to lipophilic drugs for deactivation [56,57]. UGT enzymatic activity affects the toxicity of FOLFIRNOX in pancreatic cancer patients, and inhibition of UGT-mediated glucuronidation improves the anticancer activity of gemcitabine [58–60]. Genetic polymorphism, enhanced expression, and somatic mutations of UGT genes collectively contribute to upregulated UGT activity in PDAC, that may increase production of glucuronidated products such as cholesterol glucuronide [58–61].

Individuals ≥ 50 years of age and presenting with NOD are a recently identified group at high risk of sporadic PDAC. A seminal retrospective population-based study by Chari and colleagues revealed that patients presenting with NOD have a 6–8 fold higher risk of PDAC than the general population [10,11,62]. More recent studies by Chari and colleagues demonstrated that hyperglycemia precedes PDAC diagnosis by as early as ~24–36 months [11,62]. Thus, elevations in circulating carbohydrates, such as galactosamine and glucose, may underly occult disease. Indeed, increased blood glucose are linked to diabetes and onset of PDAC [62–65]. Galactosamine is independently associated with insulin sensitivity and secretion, while circulating erythritol is associated with an increased risk of NOD [66,67]. These findings collectively demonstrate that the complementarity of microbial- and host-derived metabolites for identifying individuals at high-risk of developing PDAC.

There are considerations to the study. Information regarding chronic pancreatitis and fasting status, as well as food intake for PLCO participants was not available. Food and drink uptake were also not controlled for in the PLCO study. This is of relevance given that metabolites such as TMAO, are produced via microbial-mediated metabolism of choline, betaine, and L-carnitine which are in high amounts in animal products [21,68]. Information regarding NOD versus long-standing DM was not available for the PLCO cohort, precluding evaluation of existing risk models [11]. Information regarding changes in weight loss or changes in glycemic indices for PLCO participants were also not available. Prior studies have demonstrated that the NOD when accompanied by weight loss is substantially associated with increased risk of developing PDAC [69]. Longitudinal evaluations of the metabolite panel are warranted to ensure stability and reliability of biomarker readouts (i.e. stable in controls and stable or rising in cases) as well as to assess for potential improvements in risk prediction [70–72]. While the metabolite panel was developed to predict 5-year risk of PDAC, it remains to be determined whether predictive performance is maintained when extending beyond 5 years. Additionally, it remains to be determined whether the metabolite panel may inform upon risk of other cancer types [73]. This is a point of relevance given that microbial-associated metabolite, such as TMAO, are linked with increased risk of other GI cancers, such as colorectal cancer [74,75]. To this end, a major on-going initiative of the group is towards testing of the metabolite biomarker panel for risk prediction of PDAC individuals with NOD [76]. The study set includes serial blood specimens from participants as well as other cancer types that are prevalent among individuals with NOD that completed the three-year study follow-up period [76]. The study will also allow for opportunity to determine to what extent metabolite biomarkers may differentiate late-onset type 2 diabetes versus diabetes attributed to occult disease (referred to as Type 3c diabetes) [77].

In summary, the metabolite panel for 5-year risk prediction provides a potential means for identifying those individuals at exceptionally high-risk of developing PDAC and who would benefit from surveillance and screening, as well as from potential emerging interception strategies to reduce mortality from PDAC.
